# Aspartate aminotransferase and model for end-stage liver disease reliably predict mortality in drug-induced liver injury

**DOI:** 10.1038/s41598-026-44893-8

**Published:** 2026-04-02

**Authors:** Sabine Weber, Izabel Mircheva, Rochell Balakumar, Julian Allgeier, Didem Saka, Nirali Donga, Christian M. Lange, Alexander L. Gerbes

**Affiliations:** Department of Medicine II, LMU Klinikum, Marchioninistr. 15, 81377 Munich, Munich, Germany

**Keywords:** Acute liver failure, Drug-induced liver injury, Adverse drug reaction, Acute liver injury, Liver function test, Biomarkers, Diseases, Gastroenterology, Medical research

## Abstract

Drug-induced liver injury (DILI) is associated with high mortality risk. However, no biomarker can reliably predict outcome. In order to identify baseline parameters that are associated with mortality the data of 268 prospectively collected DILI patients were analyzed. Multivariate logistic regression and receiver operating characteristic curves were used to identify the parameters being most predictive for a fatal outcome, as defined by orthotopic liver transplantation (OLT) or death. 10.4% of patients had a fatal outcome, which was associated with higher levels of transaminases, total bilirubin, INR and model for end-stage liver disease (MELD) scores (25 vs. 12, *p* < 0.001). Multivariate analysis revealed that only MELD and aspartate aminotransferase (AST) were independently associated with a poor outcome, the MELD score in particular had an extraordinarily high c-statistic of 0.93 (95% CI: 0.87–0.97). At a cut-off of ≥ 20, MELD could predict a fatal outcome with a sensitivity and specificity of 88% and 81%. The predictive performance of the MELD score could even be enhanced by combing it with AST elevation: At a cut-off of ≥ 20 and ≥ 29.6xULN, respectively, positive and negative predictive values of 44% and 96% were observed, which were higher than for any other baseline parameter.

**Supplementary Information:**

The online version contains supplementary material available at 10.1038/s41598-026-44893-8.

## Introduction

While being a rare condition, drug-induced liver injury (DILI) is one of the leading causes of acute liver failure (ALF)^1^. ALF is a highly disastrous consequence of DILI since relevant proportions of these patients require high urgency orthotopic liver transplantation (OLT) or die. As such, the observed mortality rate ranges from 1% to 6% in population-based studies^2,3^, while it has been reported to be even higher in prospective DILI registries with numbers reaching 9% to 13%^4–6^. However, successfully predicting the outcome in idiosyncratic DILI patients has been shown to be of utmost difficulty, due to variable clinical presentation and lack of prognostic biomarkers^7,8^. As such, Hy’s law, meaning total bilirubin (TBIL) elevation of more than 2x the upper limit of normal (ULN) and alanine aminotransferase (ALT) elevation of more than 3xULN, is often used as an indicator for poor prognosis in DILI^9^. However, a low specificity is limiting predictive accuracy of Hy’s law^10,11^. With new Hy’s law and the prognostic algorithm proposed by Robles et al. novel risk assessment scores have been developed^12^. However, predictive accuracy also is limited. Moreover, these scores were evaluated for the development of ALF in DILI patients^13^, while the application in predicting a fatal outcome, i.e. OLT or death, has not been validated. Nevertheless, rapid identification of the patients at risk for a fatal outcome is of high importance, since this would allow patients to receive more intense medical attention early in the course of the disease. With an early transferal to a transplant center and evaluation for high urgency OLT outcome could eventually be improved.

Taking into consideration the limitations of the existing prognostic methods in DILI, our study aims to evaluate the predictive power of baseline liver parameters and scores in a large prospectively collected DILI cohort.

## Results

### Clinical characteristics

**Fig. 1:**
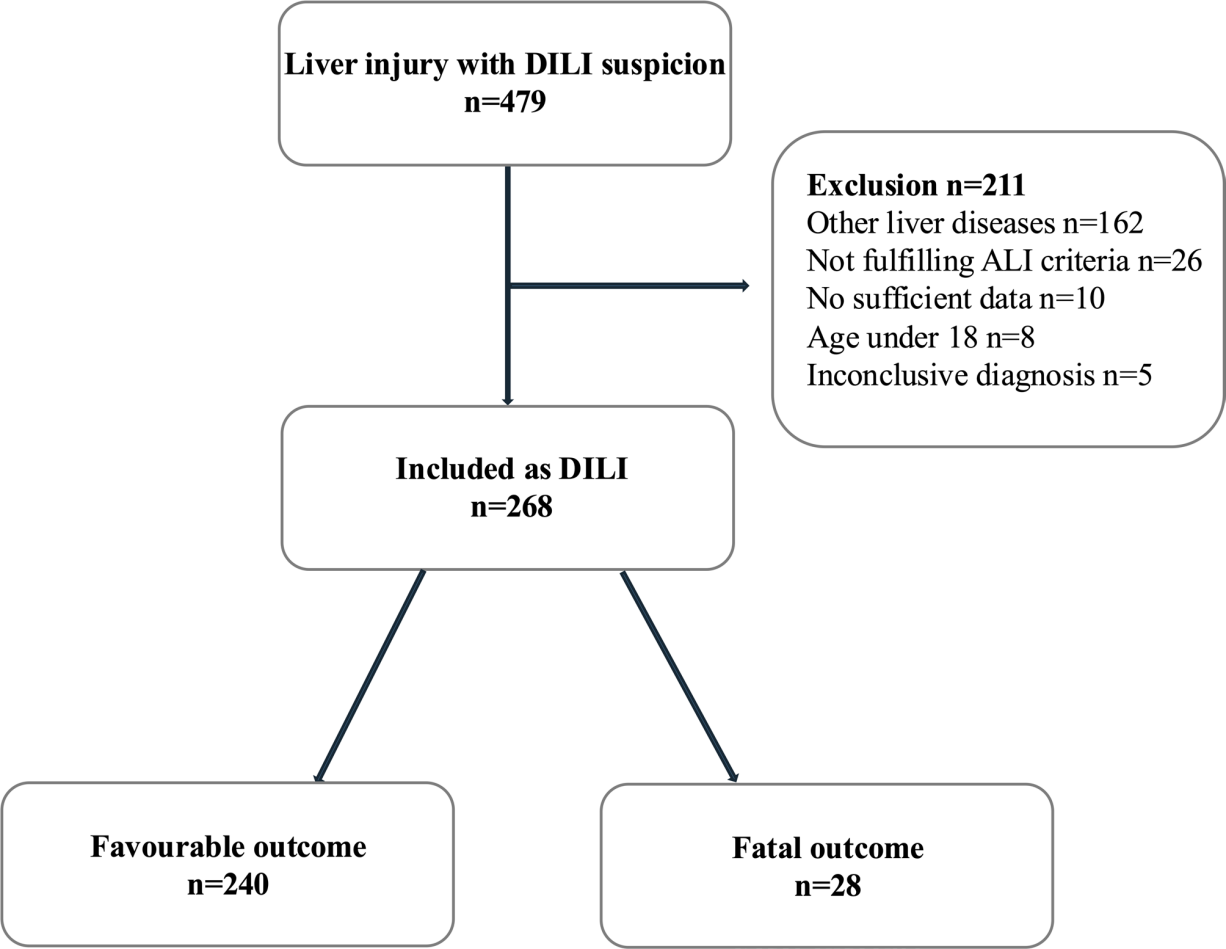
Flow chart of patient inclusion. Abbreviations: ALI: Acute liver injury; DILI: Drug-induced liver injury.

The total study population consisted of 479 patients. Only patients with idiosyncratic DILI were included in the analysis (*n* = 268). 211 patients (44.0%) were excluded: 162 due to the diagnosis alternative cause of the liver injury, 26 due to subclinical DILI not meeting the criteria for acute liver injury (ALI), 10 due to the insufficient data, eight for being under the age of 18, and five due to an inconclusive diagnosis (Fig. 1). The clinical characteristics of the 268 patients included in the analysis are displayed in Table 1. Median age was 50 years and 57.8% (*n* = 155) were female. Most patients developed DILI following the intake of analgetic (33.6%), antimicrobial (18.7%) and immunomodulating drugs (13.4%). All medications found causative for the respective DILI episode are summarized in Suppl. Table 1, organized according to their respective pharmacological or therapeutic groups. The majority of patients presented with a hepatocellular type of injury (*n* = 197, 73.5%) and had a mild or moderate DILI severity (*n* = 180, 67.2%). DILI led to ALF in 40 cases (14.9%) and 24 (60.0%) of those cases already presented with ALF at the time of DILI recognition. Jaundice was present in more than half of the patients at the time of DILI recognition (60.4%; Table 1).

**Table 1:** Clinical and laboratory characteristics.

Baseline characteristics at the time of DILI detection	
Age (years)	50 (18–84)
Body mass index (kg/m^2^)	24.2 (16.8–48.5)
Female sex	155 (57.8%)
CCI	1 (0–12)
≥ 2 drugs implicated in DILI episode	225 (84.0%)
RUCAM of the main culprit drug	6 (3–12)
Latency from beginning of main culprit drug intake until DILI recognition (days)	41 (0–2543)
R-value ^†^	14.3 (0.2- 164.6)
Type of liver injury - hepatocellular - mixed - cholestatic	197 (73.5%) 36 (13.4%) 35 (13.1%)
ALT (xULN)	20.5 (0.8–138.5.8.5)
AST (xULN)	14.1 (0.7–202.23.7.23)
AST/ALT ratio	0.68 (0.03–2.94)
ALP (xULN)	1.6 (0.4–21.0)
TBIL (mg/dl)	3.6 (0.2–40.6)
INR	1.2 (0.8–5.7)
Creatinine (mg/dl)	0.9 (0.2–7.7)
MELD	13 (6–40)
Jaundice ^a^	162 (60.4%)
Hy`s law positivity ^b^	153 (57.3%)
New Hy`s law positivity ^c^	125 (46.8%)
Prognostic algorithm by Robles et al. fulfilled ^d^	75 (28.2%)
Outcome	
Severity index - mild - moderate - severe - fatal	61 (22.8%) 119 (44.4%) 60 (22.4%) 28 (10.4%)
Coagulopathy	97 (36.2%)
Encephalopathy	44 (16.5%)
Ascites	29 (11.9%)
Acute liver failure	40 (14.9%)
Remission	199 (74.3%)
Liver transplantation	18 (6.7%)
Fatal outcome (OLT/death)	28 (10.4%)
Time between DILI recognition and OLT/death (days)	12 (1–296)

Categorical variables are presented as number and percentage (n (%)). Continuous variables are presented as median (range). ^a^ The R-value is defined as (ALT/ULN)/(ALP/ULN) with *R* ≥ 5 defining a hepatocellular, *R* ≤ 2 a cholestatic and 2 < *R* < 5 a mixed type injury. ^b^ Jaundice is defined as TBIL levels of ≥2 mg/dl at the time of the DILI detection. ^c^ Hy’s law is defined as TBIL >2xULN and ALT >3xULN. ^d^ New Hy’s law is defined as TBIL >2xULN and nR ≥ 5 with nR being ALT or AST, whichever is highest/ULN divided by ALP/ULN. ^e^ Prognostic algorithm by Robles et al. is defined as (a) AST > 17.3×ULN and TBIL > 6.6×ULN, or (b) AST ≤ 17.3xULN and AST/ALT ratio > 1.5.

Abbreviations: ALP: Alkaline phosphatase; ALT: Alanine aminotransferase; AST: Aspartate aminotransferase; CCI: Charlson Comorbidity Index; DILI: Drug-induced liver injury; MELD: Model for end-stage liver disease; RUCAM: Roussel Uclaf Causality Assessment Method; TBIL: Total bilirubin; OLT: orthotopic liver transplantation; ULN: Upper limit of normal.

### Fatal outcome – clinical and laboratory characteristics

28 patients had a fatal outcome (10.4%), out of which 18 patients received OLT (6.7%) and 10 patients died (3.7%). Median time between DILI recognition and OLT or death was 12 days (Table 1). If transplantation occurred, no donor-recipient sex matching was performed. There were no sex-specific differences within the fatal cases, out of all fatal cases 33.3% females died while 38.5% males did (*p* = 0.778). DILI-related mortality was not higher in females than males either, demonstrating that there were no sex-specific outcomes in our cohort (Table 2). Regarding other baseline characteristics, there were no differences in patients who survived or had a fatal outcome (Table 2). The burden of comorbidity, the Roussel Uclaf Causality Assessment Method (RUCAM) score of the main culprit drug and the latency between the beginning of drug intake and DILI detection in particular were comparable between the two groups. However, the rate of hepatocellular cases was higher in patients with a fatal outcome (89.3% vs. 71.1%, *p* = 0.046; Table 2). Moreover, a fatal outcome was associated with significantly higher levels of ALT, aspartate aminotransferase (AST), TBIL and INR as well as a significantly higher model for end-stage liver disease (MELD) score at baseline (25 vs. 12, *p* < 0.001). In addition, the rates of patients fulfilling the criteria for Hy’s law, new Hy’s law and the Prognostic algorithm developed by Robles et al. were all significantly higher in patients with a fatal outcome (Table 2). Baseline creatinine, however, was not associated with a fatal outcome (Table 2), demonstrating that the MELD score was mainly driven by TBIL and INR elevation. Nevertheless, four of the 28 patients with a fatal outcome had a relevant elevation in baseline creatinine of ≥ 1.50 mg/dl.

**Table 2:** Clinical and laboratory characteristics in patients with a fatal vs. favorable outcome at the time of DILI detection.

	Fatal outcome ^a^ *n* = 28	Favorable outcome *n* = 240	*p*
Age (years)	50 (19–81)	51 (18–84)	0.924
Body mass index (kg/m^2^)	24.2 (19.0–34.7.0.7)	24.2 (16.8–48.5)	0.731
Female sex	15 (53.6%)	140 (58.3%)	0.629
CCI	2 (0–12)	1 (0–10)	0.160
≥ 2 drugs implicated in DILI episode	25 (89.3%)	200 (83.3%)	0.417
RUCAM of the main culprit drug	6 (3–10)	6 (3–12)	0.821
Latency from beginning of main drug intake until DILI onset (days)	37 (0–1134)	41 (0–2543)	0.818
R-value ^b^	25.7 (0.6–83.1)	12.8 (0.2–164.6.2.6)	0.003*
Type of liver injury - hepatocellular - mixed - cholestatic	25 (89.3%) 0 (0.0%) 3 (10.7%)	172 (71.7%) 36 (15.0%) 32 (13.3%)	0.068
ALT (xULN)	45.5 (1.2–120.1.2.1)	19.6 (0.8–138.5.8.5)	< 0.001*
AST (xULN)	49.4 (1.8–202.2.8.2)	12.5 (0.7–92.5)	< 0.001*
AST/ALT ratio	1.0 (0.3–2.7)	0.6 (0.0–2.9.0.9)	< 0.001*
ALP (xULN)	1.6 (0.9–4.7)	1.6 (0.4–21.0)	0.753
TBIL (mg/dl)	14.5 (1.7–29.2)	2.7 (0.2–40.6)	< 0.001*
INR	2.1 (1.0–5.7.0.7)	1.1 (0.8–3.7)	< 0.001*
Creatinine (mg/dl)	0.9 (0.2–4.6)	0.9 (0.3–7.7)	0.322
MELD	25 (17–40)	12 (6–40)	< 0.001*
Encephalopathy	24 (85.7%)	20 (8.4%)	< 0.001*
Jaundice ^c^	27 (96.4%)	135 (56.3%)	< 0.001*
Hy`s law positivity ^d^	26 (92.9%)	127 (53.1%)	< 0.001*
New Hy`s law positivity ^e^	25 (89.3%)	100 (41.8%)	< 0.001*
Prognostic algorithm by Robles et al. fulfilled ^f^	21 (75.0%)	54 (22.7%)	< 0.001*

Categorical variables are presented as number and percentage (n (%)). Continuous variables are presented as median (range). ^a^ A fatal outcome is defined as orthotopic liver transplantation or death. ^b^ The R-value is defined as (ALT/ULN)/(ALP/ULN) with *R* ≥ 5 defining a hepatocellular, *R* ≤ 2 a cholestatic and 2 < *R* < 5 a mixed type injury. ^c^ Jaundice is defined as TBIL levels of ≥2 mg/dl at the time of the DILI detection. ^d^ Hy’s law is defined as TBIL >2xULN and ALT >3xULN. ^e^ New Hy’s law is defined as TBIL >2xULN and nR ≥ 5 with nR being ALT or AST, whichever was highest/ULN divided by ALP/ULN. ^f^ Prognostic algorithm by Robles et al. is defined as (a) AST > 17.3×ULN and TBIL > 6.6×ULN, or (b) AST ≤ 17.3xULN and AST/ALT ratio > 1.5. * indicates a statistical significance (*p* ≤ 0.05).

Abbreviations: ALP: Alkaline phosphatase; ALT: Alanine aminotransferase; AST: Aspartate aminotransferase; CCI: Charlson Comorbidity Index; DILI: Drug-induced liver injury; MELD: Model for end-stage liver disease; RUCAM: Roussel Uclaf Causality Assessment Method; TBIL: Total bilirubin; ULN: Upper limit of normal.

There were no significant differences regarding baseline characteristics and laboratory parameters between patients who underwent OLT or died (Suppl. Table 2). The only difference detected was that patients who underwent OLT were more likely to fulfill the prognostic algorithm by Robles et al. when compared to the patients who died (88.9% vs. 50.0%, *p* = 0.023), while there was no difference regarding the rate of patients being positive for Hy’s law or new Hy’s law (Suppl. Table 2).

### Baseline parameters associated with fatal outcome

In order to evaluate which parameters at baseline were associated with a fatal outcome, univariate logistic regression analysis was performed. As shown in Table 3 the R and nR values at baseline as well as a hepatocellular type of liver injury significantly correlated with a fatal outcome. Moreover, ALT, AST, the AST/ALT-ratio, TBIL, INR and the MELD score as well as the fulfilment of the criteria for Hy’s law, new Hy’s law and Prognostic algorithm by Robles et al. were all significantly associated with a fatal outcome. Interestingly, multivariate backward logistic regression analysis revealed that only AST and the MELD score were independently associated with a fatal outcome (Table 3). The MELD score in particular showed a strong correlation with the clinical outcome: the odds ratio [OR] for a fatal outcome was 1.35 per MELD point (95% confidence interval [CI] 1.20–1.52, *p* < 0.001), while the OR for AST was 1.04 (95% CI: 1.01–1.07). If the INR and MELD values of the nine phenprocoumon-induced DILI cases with INR derivation were included in the analysis, AST and MELD remained the only significant predictors for a fatal outcome, the OR for AST was 1.04 (95% CI 1.02–1.106) and the MELD score 1.26 (95% CI 1.15–1.38, *p* < 0.001) in that case (Suppl. Table 3).

**Table 3:** Logistic regression analysis regarding fatal adverse outcome in DILI.

	Univariate			Multivariate ^a^		
	OR	95% CI	*p*	OR	95% CI	*p*
Age	1.002	0.979–1.026	0.877			
Body mass index	1.001	0.920–1.090	0.978			
Male sex	1.213	0.553–2.662	0.629			
CCI	1.104	0.959–1.271	0.167			
RUCAM main culprit drug	0.972	0.764–1.237	0.818			
Latency main culprit drug	1.000	0.999–1.002	0.719			
R value	1.012	1.004–1.039	0.016*			
nR value	1.028	1.012–1.044	< 0.001*			
Hepatocellular type of liver injury	3.295	0.963–11.272	0.046*			
ALT	1.024	1.012–1.036	< 0.001*			
AST	1.052	1.033–1.072	< 0.001*	1.037	1.009–1.065	0.008*
AST/ALT ratio	4.556	2.206–9.408	< 0.001*			
ALP	0.915	0.720–1.163	0.469			
TBIL	1.151	1.091–1.214	< 0.001*			
INR	8.727	3.950–19.282.950.282	< 0.001*			
Creatinine	1.457	0.896–2.370	0.129	0.383	0.141–1.040	0.060
MELD	1.300	1.184–1.429	< 0.001*	1.349	1.195–1.523	< 0.001*
Hy`s law positivity	11.465	2.661–49.390	< 0.001*			
New Hy`s law positivity	11.583	3.403–39.425	< 0.001*	0.194	0.029–1.302	0.091
Prognostic algorithm by Robles et al.	10.222	4.125–25.334	< 0.001*			

Shown are the results of the univariate and multivariate logistic regression analysis regarding fatal outcome defined by death or orthotopic liver transplantation. ^a^ Variables considered in the multivariate analysis were baseline parameters with *p* < 0.100 in univariate analysis. Variables excluded by backward logistic regression were ALT, AST/ALT-ratio, ALP, TBIL, INR, the R and nR value, a hepatocellular type of liver injury, Hy’s law positivity, and fulfillment of the Prognostic algorithm by Robles et al. * indicates a statistical significance (*p* ≤ 0.05).

Abbreviations: ALP: Alkaline phosphatase; ALT: Alanine aminotransferase; AST: Aspartate aminotransferase; CCI: Charlson Comorbidity Index; CI: Confidence interval; DILI: Drug-induced liver injury; MELD: Model for end-stage liver disease; OR: Odds ratio; RUCAM: Roussel Uclaf Causality Assessment Method; TBIL: Total bilirubin; ULN: Upper limit of normal.

### Comparison of the predictive accuracy of baseline features

**Fig. 2:**
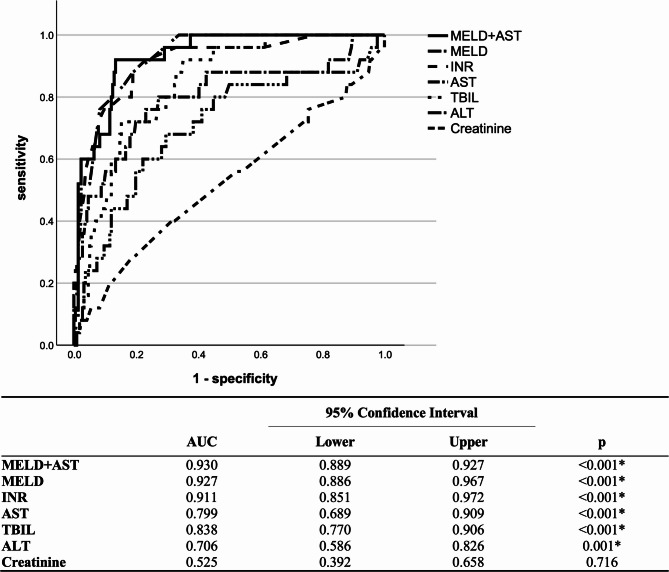
ROC analysis for fatal outcome in DILI. Shown is the ROC analysis for creatinine, ALT, TBIL, AST, INR and MELD at the time of DILI recognition. For the INR and MELD only patients without INR-derivation due to phenprocoumon were included. Abbreviations: ALT: Alanine aminotransferase; AST: Aspartate aminotransferase; DILI: Drug-induced liver injury; MELD, Model for End-Stage Liver Disease; ROC: Receiver operator characteristic; TBIL: Total bilirubin.

Receiver-operating characteristics (ROC) analysis was performed in order to identify optimal cut-off values which could best predict outcome in our DILI cohort (Fig. 2). In line with the multivariate analysis, the MELD score had the highest concordance statistic (c-statistic) for a fatal outcome (0.93, 95% CI: 0.87–0.97, *p* < 0.001), while the c-statistic for INR and AST were 0.91 and 0.80, respectively (Fig. 2). At a cut-off of 20 or higher the MELD score had a sensitivity and specificity for a fatal outcome of 88.0% and 80.9%, which was higher when compared to Hy’s law, new Hy’s law or the Prognostic algorithm by Robles et al. (Table 4). Moreover, with 98.4% the MELD score showed an extraordinarily high negative predictive value (NPV) for a fatal outcome. In addition, while being relatively low (35.9%), the positive predictive value (PPV) was still the highest for the MELD score among all baseline features (Table 4). Interestingly, the highest predictive power was observed when MELD and AST were combined (c-statistic: 0.93, Fig. 2). The sensitivity and specificity of combined model of MELD and AST at a cut-off of 0.127 were 92.0% and 86.6%, while PPV and NPV were 45.3% and 99.0% (Table 4). Moreover, PPV and NPV remained high (43.6% and 96.0%) in case the individual cut-off values for the MELD score (≥ 20) and AST (≥ 29.6xULN) were combined (Table 4). The sensitivity and specificity of the combined model of MELD and AST persisted to be extraordinarily high if the phenprocoumon cases were included (92.0% and 86.6% at a cut-off of 0.118; Suppl. Table 4). For the combination of individual cut-off values (MELD ≥ 23 and AST ≥ 29.6xULN) sensitivity was lower (60.7%), specificity (94.9%), PPV (58.6%) and NPV (95.3%), however, remained high (Suppl. Table 4).

**Table 4:** Predictive power of baseline parameters and scores regarding a fatal outcome in DILI.

	Cut-off	Sensitivity	Specificity	PPV	NPV
Hy’s law positivity		92.9%	46.9%	28.0%	96.3%
New Hy’s law positivity		89.3%	58.2%	20.0%	97.9%
Prognostic algorithm by Robles et al.		75.0%	77.3%	17.0%	98.2%
Kings College Criteria		78.6%	98.8%	88.0%	97.5%
Creatinine	1.1	28.0%	81.4%	18.0%	91.1%
ALT	36.0	68.0%	70.2%	21.8%	95.0%
TBIL	12.1	72.0%	84.7%	35.7%	96.2%
AST	29.6	76.0%	76.6%	28.0%	96.4%
INR	1.4	88.0%	80.9%	32.0%	98.9%
MELD	20	88.0%	80.9%	35.9%	98.4%
MELD-AST model (logistic regression model)	0.127	92.0%	86.6%	45.3%	99.0%
MELD & AST (individual cut-offs)	20 & 29.6	65.4%	90.8%	43.6%	96.0%

This table reflects the optimal cut-off values as well as the sensitivity and specificity, positive and negative predictive values of baseline parameters and scores regarding the prediction of a fatal outcome in DILI patients. A fatal outcome was defined by orthotopic liver transplantation or death. The cut-off values were determined by ROC curve analysis and Youden’s index.

Abbreviations: ALT: Alanine aminotransferase; AST: Aspartate aminotransferase; CI: Confidence interval; c-statistics: Concordance statistic; INR: International normalized ratio; MELD: Model for end-stage liver disease; NPV: Negative predictive value; PPV: Positive predictive value; ROC: Receiver operator characteristics; TBIL: Total bilirubin; ULN: Upper limit of normal.

Since the Kings College Criteria (KCC) are used for allocation for high urgency OLT, the fulfillment of KCC was also evaluated in our cohort. KCC were fulfilled in 25 patients (9.3%) in total, and the predictive accuracy was relatively high with a PPV of 88.0% and a NPV of 97.5%; however, with only 78.6% the sensitivity was lower than compared to the MELD score or the combination of MELD and AST (Table 4). Moreover, KCC were mostly not fulfilled at the time of DILI recognition, only three patients (12.0%) presented with positive KCC at the time of onset, while the median time to fulfilment was 6 days (range: 0–45 days, data not shown).

## Discussion

The severity of DILI can range from asymptomatic elevation of aminotransferases to fulminant liver failure leading to high urgency OLT or death. Various studies have looked at risk factors for severe DILI, showing that female sex, hepatocellular damage, higher levels of ALT, TBIL, or INR, Asian or African American race, itching, concomitant lung diseases, hypalbuminemia and a low platelet count are associated with a more severe form of DILI^4,10,14,15^. Moreover, certain histological changes such as necrosis, higher fibrosis stages, microvesicular steatosis, ductular reaction and bile duct loss are more frequently found in severe DILI cases^16,17^, while the presence of eosinophilia or lipofuscinosis is related to a more favorable outcome in DILI^17,18^. However, what is needed in clinical practice are laboratory parameters with specific cut-off points, which can direct clinicians to whether the individual patient is at risk for a fatal outcome or not. In clinical practice, Hy’s law is widely used^19^, however the prognostic accuracy is limited, expressed in particular by a low PPV and a limited specificity^10^, which is also demonstrated in our cohort: While Hy’s law had a sensitivity for predicting OLT or death of 93%, specificity was low with only 47% and the PPV was only 28%.

In order to overcome these limitations, Robles-Diaz et al. developed the new Hy’s law, which is defined as the ratio of ALT or AST, whichever is the highest, and alkaline phosphatase (ALP) combined with jaundice (TBIL>2xULN)^12^. In our cohort, new Hy’s law had a similarly high sensitivity of 89% for predicting a fatal outcome when compared to Hy’s law, however, specificity was only slightly higher with 58%. Moreover, PPV was even lower with only 20%, which is in line with previous data reporting a PPV of only 12% for a two-year DILI-related mortality for the new Hy’s law^10^. Moreover, a composite algorithm was proposed by Robles et al. which has been shown to indicate higher risk for ALF or OLT in case AST is higher than 17.3xULN and TBIL is higher than 6.6xULN or the AST/ALT-ratio is above 1.5 in the cases with an AST below 17.3xULN^12^. In our cohort, the specificity of this algorithm was higher than compared with Hy’s law and new Hy’s law (77%), however, sensitivity was compromised with only 75%. Thus, we could not replicate the high sensitivity and specificity of 82% and 80% which had initially been reported^12^. In addition, with only 17% the PPV of this composite algorithm was even lower when compared to Hy’s law and new Hy’s law in our cohort. The differences in the predictive power of the algorithm observed by us and Robles et al. might be due to the diverging endpoints. Robles et al. evaluated the predictive accuracy of their algorithm with regards to ALF and OLT, while we chose OLT or death as the primary endpoint. ALF, however, is not an optimal outcome measure in DILI cases, since ALF is often already present at the initial presentation, in particular in more fulminant cases. This is also reflected by our data, showing that 60% of the ALF patients already fulfilled ALF criteria at the time of DILI recognition.

Interestingly, multivariate analysis revealed that only AST and the MELD score were independently associated with DILI-related mortality in our cohort. The MELD score in particular showed a high OR of 1.35 per MELD point and an extraordinarily high c-statistic of 0.93. At a cut-off of 20, the MELD score had a sensitivity and specificity for a fatal outcome of 88% and 81%, respectively, while the NPV was 98%.

The MELD score was mainly driven by INR and TBIL elevation in our cohort, while a relevant increase in creatinine contributed to the elevation of the MELD score in four of the 28 fatal cases. These findings highlight the clinical relevance of the MELD score, as it integrates both hepatic dysfunction and renal impairment, thereby providing a comprehensive measure of disease severity across different stages of clinical deterioration.

The predictive power of the MELD score regarding early DILI-related mortality has been evaluated in a few studies before. In a retrospective analysis from Seoul, it was shown that MELD and hemoglobin were independent predictors of poor outcome. Regarding a 30-day mortality, the c-statistic for MELD was 0.93 with an optimal cut-off at 20.5^6^. In a large study by the US American DILI network, it was shown that the MELD score at a cut-off of 19 had a c-statistic of 0.83 for early DILI fatalities, which was higher than for Hy’s law (0.60) or new Hy’s law (0.73)^10^. In a cohort of 82 Indian patients, encephalopathy, MELD score and ALP, each measured one week after drug discontinuation, independently predicted mortality^20^. In this study, however, encephalopathy showed a higher association with the mortality risk than the MELD score (OR 88.7 vs. 5.5)^20^. Furthermore, in clinical practice it is not always feasible to wait for one week in order to perform a risk assessment, particularly in the more fulminant ALF cases. More recently, the predictive performance of the MELD score was reported by a German tertiary center, demonstrating that a MELD score at a cut-off of 18 had a sensitivity and specificity of 88% and 72% for a poor outcome^21^.

Strikingly, our current analysis showed that the predictive power of the MELD score could even be enhanced by combining it with AST. The c-statistic for a combined MELD-AST-model was 0.93, while sensitivity and specificity were as high as 92% and 87%. Out of all baseline parameters the combination of MELD being 20 or higher and AST being at least 29.6xULN had the highest PPV (44%) for a fatal outcome with a remarkably high NPV (96%).

While it is commonly believed that the level of aminotransferase elevation does not correlate with prognosis, we could show an association of highly elevated AST levels with DILI-related mortality. Moreover, even though ALT is generally considered more liver-specific than AST and ALT but not AST is part of the ALI definition used for patient inclusion in DILI studies^22^, AST demonstrated a stronger association with clinical outcomes in our cohort. Nevertheless, our findings are also in line with some earlier studies in DILI patients^12,23^. A pathophysiological explanation for the association of AST elevation with a poorer prognosis in DILI might be that a drastic increase in AST demonstrates massive hepatocytic damage and necrosis eventually leading to the destruction of mitochondrial integrity and therefore the release of AST from mitochondria.

KCC performed relatively well in our cohort with regards to prediction of DILI-related mortality. Yet, sensitivity was lower than for the MELD score and the fulfilment of KCC was mostly not given at the time of DILI recognition, but further on during the hospitalization. Thus, KCC are not helpful in identifying the patients at risk for a fatal outcome early during the course of the disease. When positive, however, KCC indicate a severely high mortality risk, which therefore justifies that high urgency OLT listing is pursued.

Interestingly, we could not identify any influence of the burden of comorbidities on outcome in our DILI cohort. This is in contrast to a previous study by the DILI network which demonstrated that a model comprised of a significant comorbidity (defined as a Charlson comorbidity index [CCI] > 2), the MELD score and albumin was highly associated with six-month all-cause mortality following DILI. Moreover, significant comorbidity was associated with mortality in DILI patients both in individuals with a MELD ≤ 19 as well as > 19 showing that comorbidity by itself was a risk factor for mortality following DILI^24^. Differences in the study design with diverging approaches to risk estimation might be the reason for the different findings.

Strengths of our study include the prospective study design, the large sample size and the thorough work-up of each individual patient with an exclusion of all potential non-DILI cases. There are several limitations to our study, in particular the lack of an external validation cohort and the retrospective design of the data analysis. However, the latter is an inherent problem in DILI-related studies since DILI diagnosis is largely based on exclusion of alternative causes and evolution upon long-term follow-up^25^. Moreover, in comparison to other registry studies, we here present a single-center study from a tertiary center, which could have contributed to the higher proportion of severe cases in our cohort. Nevertheless, we could confirm in our large and prospectively collected cohort of DILI patients, that the MELD score can predict DILI-related mortality with high accuracy. Moreover, the combination of the MELD score and a relevant AST elevation had an even higher predictive power. Our results could therefore help clinical decision making and with this possibly enhance clinical outcome in fulminant DILI cases. To overcome the limitations of a single-center study with a possible selection bias towards more severe cases, our results should be validated externally in prospective and preferably multi-centric DILI cohort. In addition, it would be of interest if our observations also apply to patients with ALI caused by hepatotropic viruses or alcohol.

In conclusion, we propose that the combination of a MELD score at a cut-off of ≥ 20 and AST at a cut-off of ≥ 29.6xULN predicts a high mortality risk in DILI patients and should prompt a transferal to a transplant center and rapid evaluation for OLT. To the best of our knowledge, this is the first study demonstrating that the combination of the MELD score with a significant elevation in AST can predict DILI-related mortality.

## Methods

### Study cohort

The analysis is based on patients who were recruited for our prospective study on ALI with potential drug-induced cause between 2012 and 2024 at the LMU Klinikum in Munich, Germany. The study protocol, inclusion and exclusion criteria have been described in more detail in an earlier study^26^. The present study represents an extension of this cohort. Patient recruitment and data collection were continued using the same inclusion criteria and standardized assessment protocols. All procedures were in accordance with the Helsinki Declaration of 1975, as revised in 2013 and the study protocol was approved by the local ethics committee (Project number 55 − 13). Written informed consent was obtained from all subjects.

ALI was defined according to international consensus criteria^22^: a) ALT ≥ 5×ULN, (b) ALP ≥ 2×ULN or (c) ALT ≥ 3×ULN and TBIL ≥ 2×ULN. In addition, *n* = 3 patients were included due to clinical diagnosis of DILI and TBIL elevation ≥5xULN together with an elevation of aminotransferases. The type of liver injury was classified using the R-ratio values, (ALT/ULN)/(ALP/ULN), with *R* ≥ 5 defining a hepatocellular, *R* ≤ 2 a cholestatic and 2 < *R* < 5 a mixed type of injury^22^. As baseline liver parameters the first available blood tests indicating DILI were utilized, in most cases this was at the time of ALI diagnosis, while in a minority of cases liver injury was already detectable before ALI criteria were fulfilled. The R-ratio was also calculated using the first available blood test when liver injury was detected.

### Data acquisition

Clinical and laboratory data was acquired from thorough interrogation of the patients as well as from the hospital database and included demographics, all drugs taken in the last 12 months before liver injury, the latency between the beginning of drug intake and the onset of ALI, concomitant diseases, clinical symptoms, as well as laboratory, serological, imaging and histopathological results. In addition, the evolution of liver parameter elevation was evaluated for as long as possible for each patient. These follow-up data were retrieved through the hospital database and additional telephone consultations in case the patients did not present at the hospital for regular follow-up appointments.

To objectively assess the comorbidities, the CCI, a tool for estimating 1-year all-cause mortality risk, was evaluated for each patient^27^. The CCI is calculated based on the presence or absence of 17 comorbidities including myocardial infarction, dementia, peptic ulcer disease, and solid malignancy among others^27^.

### DILI diagnosis and exclusion of alternative causes

A thorough hepatological work-up was performed for every patient including virology testing, hepatic imaging, testing for autoantibodies and for metabolic as well as hereditary liver diseases. While every patient initially enrolled in our study was suspected to suffer from DILI, only patients with the final diagnosis of idiosyncratic DILI were included in the current analysis. Diagnosis of idiosyncratic DILI was based on clinical, laboratory and histopathologic findings, the RUCAM score^28^, the exclusion of alternative causes for liver injury, causality assessment by the physician in charge and upon long-term follow-up. A significant proportion of the patients (198/479, 41.3%) was additionally evaluated in a structured expert opinion causality case assessment process^29^. Alternative causes for ALI which led to the exclusion of patients were viral hepatitis, autoimmune hepatitis, autoimmune cholestatic liver diseases, metabolic-dysfunction associated steatotic liver disease, alcoholic liver disease, hereditary liver diseases, biliary obstruction, malignant infiltration of the liver and intrinsic DILI caused by acetaminophen.

### Definition of severity

Hy`s law was defined as TBIL>2xULN and ALT>3xULN at the time of DILI recognition^9^. New Hy`s law was defined as TBIL>2xULN and nR ≥ 5 with nR being ALT or AST, whichever was highest/ULN divided by ALP/ULN^12^. The prognostic algorithm by Robles et al. is supposed to identify patients with high risk for ALF and/or OLT by AST > 17.3×ULN and TBIL > 6.6×ULN, while in patients with AST ≤ 17.3xULN those with an AST/ALT ratio > 1.5 are at higher risk^12^. The fulfilment of the KCC for non-paracetamol-induced liver failure was evaluated for every patient with ALF^30^. The MELD score, which has been developed to evaluate the severity of advanced chronic liver disease and is based on TBIL, serum creatinine, and INR^31^, was computed for each DILI patient at the time of DILI recognition with values ranging from 6 to 40. The formula used to calculate the MELD score was: MELD(i) = 0.957 × ln(Cr) + 0.378 × ln(bilirubin) + 1.120 × ln(INR) + 0.643)^32^. Patients with phenprocoumon-induced DILI (*n* = 9) were excluded from primary INR and MELD analyses in case a drug-induced INR elevation was detectable. ALF was defined according to international criteria: (1) absence of pre-existing liver disease, (2) coagulopathy with an INR (International Normalized Ratio) ≥ 1.5 in the absence of oral anticoagulants, and (3) hepatic encephalopathy^33^. A fatal outcome was defined as OLT or DILI-related death.

### Statistical analysis

All statistical analyses were conducted using IBM SPSS Statistics (version 29.0.2.0). Categorical variables were reported as number and percentages, while continuous variables were presented as median and range. Categorical variables were compared using the Chi-square test, while the Mann-Whitney U test was applied for continuous variables. *p* ≤ 0.05 was considered statistically significant. In order to evaluate the prognostic power of baseline parameters, univariate and multivariate logistic regression, the latter with a stepwise backward elimination, were performed. Only variables with p-values < 0.1 in the univariate analysis were included in the multivariate analysis. A ROC curve was utilized to identify the c-statistic of baseline values with regards to the prediction of a fatal outcome. Cut-off values for the parameters with prognostic importance were established using the Youden`s index. Furthermore, sensitivity, specificity, the PPV and NPV were calculated. In addition, the predicted probability of a fatal outcome was retrieved from a logistic regression model comprised of the two independent predictors of a fatal outcome, i.e. MELD and AST. Since for practical reasons it is more feasible to work with well-established cut-off values, the analysis of the predictive power of the combination of MELD and AST was done for the combined score derived from the logistic regression model as well as for the combination of the individual cut-off values.

## Supplementary Information

Below is the link to the electronic supplementary material.

Supplementary Material 1

Supplementary Material 2

Supplementary Material 3

Supplementary Material 4

## Data Availability

All data generated or analysed during this study are included in this article. Further enquiries can be directed to the corresponding author.
