# Supplementary material for: Aspartate aminotransferase and model for end-stage liver disease reliably predict mortality in drug-induced liver injury
Source: Sci Rep. 2026 Apr 2;16:11236. doi: 10.1038/s41598-026-44893-8 (PMC13047038; doi:10.1038/s41598-026-44893-8)
Supplement: Supplementary file 1 — Supplementary Material 1 [file 41598_2026_44893_MOESM1_ESM.docx]

**Suppl. Table 2 Clinical and laboratory characteristics in patients undergoing liver transplantation vs. those who died**

|  | **OLT**  **n=18** | **Death**  **n=10** | **p** |
| --- | --- | --- | --- |
| **Age (years)** | 48 (19-65) | 72 (27-81) | 0.236 |
| **Body mass index (kg/m^2^)** | 23.2 (20.1-34.7) | 25.3 (19.0-31.0) | 1.000 |
| **Female sex** | 8 (44.4%) | 5 (50.0%) | 0.778 |
| **CCI** | 1 (0-5) | 4 (0-12) | 0.236 |
| **≥ 2 drugs implicated in DILI episode** | 16 (88.9%) | 9 (90.0%) | 0.927 |
| **RUCAM of the main culprit drug** | 7 (3-10) | 6 (4-7) | 0.434 |
| **Latency from beginning of main drug intake until DILI onset (days)** | 34 (5-1134) | 40 (0-455) | 0.695 |
| **R-value ^a^** | 29.0 (1.0-83.1) | 15.0 (0.6-58.9) | 0.236 |
| **Type of liver injury**   - **hepatocellular** - **mixed** - **cholestatic** | 17 (94.4%)  0 (0.0%)  1 (5.6%) | 8 (80.0%)  0 (0.0%)  2 (20.0%) | 0.236 |
| **ALT (xULN)** | 56.3 (1.2-120.1) | 33.0 (1.6-108.2) | 0.236 |
| **AST (xULN)** | 53.5 (2.7-202.2) | 37.8 (1.8-115.2) | 0.695 |
| **AST/ALT ratio** | 0.95 (0.31-2.68) | 1.08 (0.69-1.82) | 0.236 |
| **ALP (xULN)** | 1.5 (1.0-4.1) | 2.1 (0.9-4.7) | 0.695 |
| **TBIL (mg/dl)** | 16.1 (6.6-29.2) | 9.9 (1.7-23.6) | 0.236 |
| **INR** | 2.2 (1.3-5.3) | 1.7 (1.0-5.7) | 0.695 |
| **Creatinine (mg/dl)** | 0.9 (0.6-1.9) | 0.9 (0.2-4.6) | 1.000 |
| **MELD** | 26 (19-40) | 23 (17-37) | 0.705 |
| **Jaundice ^b^** | 18 (100.0%) | 9 (90.0%) | 0.172 |
| **Encephalopathy** | 17 (94.4%) | 7 (70.0%) | 0.077 |
| **Ascites** | 7 (38.9%) | 5 (50.0%) | 0.569 |
| **Hy`s law positivity ^c^** | 17 (94.4%) | 9 (90.0%) | 0.662 |
| **New Hy`s law positivity ^d^** | 17 (94.4%) | 8 (80.0%) | 0.236 |
| **Prognostic algorithm by Robles et al. fulfilled ^e^** | 16 (88.9%) | 5 (50.0%) | **0.023*** |

Categorical variables are presented as number and percentage (n (%)). Continuous variables are presented as median (range). ^a^ The R-value is defined as (ALT/ULN)/(ALP/ULN) with R ≥ 5 defining a hepatocellular, R ≤ 2 a cholestatic and 2 < R < 5 a mixed type injury. ^b^ Jaundice is defined as TBIL levels of ≥2mg/dl at the time of the DILI detection. ^c^ Hy’s law is defined as TBIL >2xULN and ALT >3xULN. ^d^ New Hy’s law is defined as TBIL >2xULN and nR ≥5 with nR being ALT or AST, whichever was highest/ULN divided by ALP/ULN. ^e^ Prognostic algorithm by Robles et al. is defined as a) AST > 17.3×ULN and TBIL> 6.6×ULN, or b) AST≤17.3xULN and AST/ALT ratio > 1.5. * indicates a statistical significance (p≤0.05).

Abbreviations: ALP: Alkaline phosphatase; ALT: Alanine aminotransferase; AST: Aspartate aminotransferase; CCI: Charlson Comorbidity Index; DILI: Drug-induced liver injury; MELD: Model for end-stage liver disease; OLT: Orthotopic liver transplantation; RUCAM: Roussel Uclaf Causality Assessment Method; TBIL: Total bilirubin; ULN: Upper limit of normal.
