# Supplementary material for: Aspartate aminotransferase and model for end-stage liver disease reliably predict mortality in drug-induced liver injury
Source: Sci Rep. 2026 Apr 2;16:11236. doi: 10.1038/s41598-026-44893-8 (PMC13047038; doi:10.1038/s41598-026-44893-8)
Supplement: Supplementary file 2 — Supplementary Material 2 [file 41598_2026_44893_MOESM2_ESM.docx]

**Suppl. Table 3 Logistic regression analysis regarding fatal adverse outcome including phenprocoumon-induced liver injury cases**

|  | **Univariate** | | | **Multivariate ^a^** | | |
| --- | --- | --- | --- | --- | --- | --- |
|  | **OR** | **95 % CI** | **p** | **OR** | **95 % CI** | **p** |
| **Age** | 1.002 | 0.979-1.026 | 0.877 |  |  |  |
| **Body mass index** | 1.001 | 0.920-1.090 | 0.978 |  |  |  |
| **Male sex** | 1.213 | 0.553-2.662 | 0.629 |  |  |  |
| **CCI** | 1.104 | 0.959-1.271 | 0.167 |  |  |  |
| **RUCAM main culprit drug** | 0.972 | 0.764-1.237 | 0.818 |  |  |  |
| **Latency main culprit drug** | 1.000 | 0.999-1.002 | 0.719 |  |  |  |
| **R value** | 1.012 | 1.004-1.039 | **0.016*** | 0.975 | 0.950-1.000 | 0.051 |
| **nR value** | 1.028 | 1.012-1.044 | **<0.001*** |  |  |  |
| **Hepatocellular type of liver injury** | 3.295 | 0.963-11.272 | **0.046*** |  |  |  |
| **ALT** | 1.024 | 1.012-1.036 | **<0.001*** |  |  |  |
| **AST** | 1.052 | 1.033-1.072 | **<0.001*** | 1.039 | 1.016-1.062 | **<0.001*** |
| **AST/ALT ratio** | 4.556 | 2.206-9.408 | **<0.001*** |  |  |  |
| **ALP** | 0.915 | 0.720-1.163 | 0.469 |  |  |  |
| **TBIL** | 1.151 | 1.091-1.214 | **<0.001*** |  |  |  |
| **INR** | 5.041 | 2.937-8.654 | **<0.001*** |  |  |  |
| **Creatinine** | 1.457 | 0.896-2.370 | 0.129 |  |  |  |
| **MELD** | 1.274 | 1.175-1.381 | **<0.001*** | 1.256 | 1.48-1.375 | **<0.001*** |
| **Hy`s law positivity** | 11.465 | 2.661-49.390 | **<0.001*** |  |  |  |
| **New Hy`s law positivity** | 11.583 | 3.403-39.425 | **<0.001*** |  |  |  |
| **Prognostic algorithm by Robles et al.** | 10.222 | 4.125-25.334 | **<0.001*** |  |  |  |

Shown are the results of the univariate and multivariate logistic regression analysis regarding fatal outcome defined by death or orthotopic liver transplantation. Included in this analysis were the INR and MELD scores of all patients, including those with phenprocoumon-induced DILI. ^a^Variables considered in the multivariate analysis were baseline parameters with p<0.100 in univariate analysis. Variables excluded by backward logistic regression were ALT, AST/ALT-ratio, ALP, creatinine, TBIL, INR, a hepatocellular type of liver injury, Hy’s law positivity, new Hy’s law positivity and fulfillment of the Prognostic algorithm by Robles et al. * indicates a statistical significance (p≤0.05).

Abbreviations: ALP: Alkaline phosphatase; ALT: Alanine aminotransferase; AST: Aspartate aminotransferase; CCI: Charlson Comorbidity Index; CI: Confidence interval; DILI: Drug-induced liver injury; MELD: Model for end-stage liver disease; OR: Odds ratio; RUCAM: Roussel Uclaf Causality Assessment Method; TBIL: Total bilirubin; ULN: Upper limit of normal.
