# Supplementary material for: Aspartate aminotransferase and model for end-stage liver disease reliably predict mortality in drug-induced liver injury
Source: Sci Rep. 2026 Apr 2;16:11236. doi: 10.1038/s41598-026-44893-8 (PMC13047038; doi:10.1038/s41598-026-44893-8)
Supplement: Supplementary file 3 — Supplementary Material 3 [file 41598_2026_44893_MOESM3_ESM.docx]

**Suppl. Table 1 Drug classes causative for DILI**

| **Culprit drug class** | **N (%)** |
| --- | --- |
| **Analgetics** | 90 (33.6%) |
| **Antimicrobials** | 50 (18.7%) |
| **Immunomodulators** | 36 (13.4%) |
| **Herbal and dietary supplements** | 23 (8.6%) |
| **Centrally acting agents** | 15 (5.6%) |
| **Oral anticoagulation and antiplatelet agents** | 11 (4.1%) |
| **Checkpoint inhibitors** | 9 (3.4%) |
| **Statins** | 8 (3,0%) |
| **Cardiovascular drugs** | 7 (2.6%) |
| **Endocrinological** | 6 (2.2%) |
| **Anti-tumor agents** | 5 (1.9%) |
| **Proton pump inhibitors** | 4 (1.5%) |
| **Others** | 4 (1.5%) |

Variables are presented as number and percentage (n (%)).

The medications categorized by these groups are the following:

^a^ Analgetics: Diclofenac, ibuprofen, indomethacin, metamizole, naproxen, paracetamol, sumatriptan

^b^ Antimicrobials: Acyclovir, albendazole, amoxicillin, amoxicillin/clavulanic acid, ampicillin/sulbactam, azithromycin, cefpodoxime, ceftriaxone, cefuroxime, ciprofloxacin, clindamycin, co-trimoxazole, doxycycline, flucloxacillin, fluconazole, fosfomycin, isoniazid, itraconazole, mebendazole, metronidazole, minocycline, moxifloxacin, nitrofurantoin, piperacillin/tazobactam, rifampicin

^c^ Immunomodulators: Anakinra, azathioprine, dimethyl fumarate, fingolimod, infliximab, leflunomide, methylprednisolone, natalizumab, pirfenidone

^d^ Herbal and dietary supplements: Ashwagandha, 6-bromoandrostenedione, BAOWOW, Canephron, chelidonium, chlorella, chondroitin, devil’s claw, flavonoids, Grippostad, Iberogast, Mivolis Immune Complex, multivitamins, nabiximols, Potaba-Glenwood, silymarin, St. John’s wort, Synergon cicuta virosa, turmeric.

^e^ Oral anticoagulation and antiplatelet agents: Apixaban, clopidogrel, dabigatran, phenprocoumon, prasugrel, rivaroxaban

^f^ Centrally acting agents: aripiprazole, carbamazepine, desflurane, flupirtine, fluspirilene, gabapentin, lacosamide, levetiracetam, opipramol, oxomemazine, pregabalin, sertraline, sevoflurane

^g^ Checkpoint inhibitors: Atezolizumab, ipilimumab, nivolumab, pembrolizumab, tremelimumab

^h^ Statins: Atorvastatin, simvastatin

^i^ Cardiovascular drugs: Amiodarone, digoxin, ezetimibe, methyldopa

^j^ Endocrinological drugs: Alendronate, carbimazole, ethinylestradiol

^k^ Anti-tumor agents: Bortezomib, enzalutamide, imatinib, sunitinib

^l^ Proton pump inhibitors: Esomeprazole, omeprazole, pantoprazole

^m^ Others: Acitretin, cetirizine, COVID-19 vaccination, rupatadine
