# Supplementary material for: Aspartate aminotransferase and model for end-stage liver disease reliably predict mortality in drug-induced liver injury
Source: Sci Rep. 2026 Apr 2;16:11236. doi: 10.1038/s41598-026-44893-8 (PMC13047038; doi:10.1038/s41598-026-44893-8)
Supplement: Supplementary file 4 — Supplementary Material 4 [file 41598_2026_44893_MOESM4_ESM.docx]

**Suppl. Table 4 Predictive power of baseline parameters and scores regarding a fatal outcome in DILI including phenprocoumon-induced liver injury cases**

|  | **Cut-off** | **Sensitivity** | **Specificity** | **PPV** | **NPV** |
| --- | --- | --- | --- | --- | --- |
| **Hy’s law positivity** |  | 92.9% | 46.9% | 28.0% | 96.3% |
| **New Hy’s law positivity** |  | 89.3% | 58.2% | 20.0% | 97.9% |
| **Prognostic algorithm by Robles et al.** |  | 75.0% | 77.3% | 17.0% | 98.2% |
| **Kings College Criteria** |  | 78.6% | 98.8% | 88.0% | 97.5% |
| **Creatinine** | 1.1 | 28.0% | 81.4% | 18.0% | 91.1% |
| **ALT** | 36.0 | 68.0% | 70.2% | 21.8% | 95.0% |
| **TBIL** | 12.1 | 72.0% | 84.7% | 35.7% | 96.2% |
| **AST** | 29.6 | 76.0% | 76.6% | 28.0% | 96.4% |
| **INR** | 1.4 | 89.3% | 79.5% | 31.3% | 98.9% |
| **MELD** | 23 | 78.6% | 91.5% | 52.4% | 97.3% |
| **MELD-AST model (logistic regression model)** | 0.118 | 92.0% | 86.6% | 44.1% | 99.0% |
| **MELD & AST (individual cut-offs)** | 23 & 29.6 | 60.7% | 94.9% | 58.6% | 95.3% |

This table reflects the optimal cut-off values as well as the sensitivity and specificity, positive and negative predictive values of baseline parameters and scores regarding the prediction of a fatal outcome in DILI patients including the INR and MELD analyses of the cases with phenprocoumon-induced INR derivation. A fatal outcome was defined by orthotopic liver transplantation or death. The cut-off values were determined by ROC curve analysis and Youden’s index.

Abbreviations: ALT: Alanine aminotransferase; AST: Aspartate aminotransferase; CI: Confidence interval; c-statistics: Concordance statistic; INR: International normalized ratio; MELD: Model for end-stage liver disease; NPV: Negative predictive value; PPV: Positive predictive value; ROC: Receiver operator characteristics; TBIL: Total bilirubin; ULN: Upper limit of normal.
